# Molecular Resonance Quantification and Label‐Free Interactome Characterization of Total Proteome of Tumor Specimens Decipher Responder and Success Predictors in Colorectal Cancer Patients Treated With Panitumumab

**DOI:** 10.1002/cam4.71387

**Published:** 2025-11-30

**Authors:** Angelique Quartier, Ahmed Y. Sanin, Julia Nagelschmitz, Justine Schneider, Wenjie Shi, Thomas Wartmann, Maximilian Dölling, Frederike Stelter, Mihailo Andric, Roland S. Croner, Pierre Eftekhari, Ulf D. Kahlert

**Affiliations:** ^1^ Inoviem Scientific Illkirch Graffenstaden France; ^2^ Molecular and Experimental Surgery, University Clinic for General, Visceral, Vascular and Transplantation Surgery, Medical Faculty University Hospital Magdeburg Otto‐von Guericke University Magdeburg Germany; ^3^ Research Campus STIMULATE Otto von Guericke University Magdeburg Magdeburg Germany

**Keywords:** biomarkers, colorectal cancer (CRC), EGFR signaling pathway, proteomics

## Abstract

**Background:**

Panitumumab shows limited clinical benefit in colorectal cancer (CRC), and reliable predictive biomarkers to guide patient selection remain lacking. To address this gap, we investigated molecular determinants of therapeutic response using tumor samples from patients with primary and metastatic CRC. By integrating PIMS‐based metastatic classification, NPOT interaction profiling and quantitative proteomics, this study aimed to identify response‐associated pathways and potential prognostic biomarkers that could support improved stratification for panitumumab therapy.

**Method:**

Twenty‐one tumor resection samples from twenty CRC patients from primary site (*n* = 12) and from intrahepatic metastasis (*n* = 9), female (*n* = 6) and male (*n* = 14) were analyzed. Clinical metadata of donors was associated with molecular properties of each sample. Patients’ cryostored tumor material was blinded before subjecting to PIMS analysis. PIMS analysis was at first performed to separate metastatic and non‐metastatic patients. After uncovering the blind, tumors were all challenged by 1 μg of panitumumab in PIMS to identify responder and non‐responder with or without metastasis. Tumors from metastatic (*n* = 3) and non‐metastatic (*n* = 2) patients were thereafter analyzed by NPOT to identify EGFR‐related signaling pathway. All group tumors were analyzed using label‐free quantitative proteomics.

**Results:**

PIMS identified with 82% accuracy metastatic (*n* = 9) from non‐metastatic (*n* = 7) tumor. The metastatic tumor had higher resonance volumes (2948–5094) compared to non‐metastatic (1076–2759) tumor. NPOT identified EGFR only in metastatic tumor. The metastatic interactome was composed of 34 proteins (EGFR, PTPN1, CTNNB1, CTNND1, YWHAZ, CD44, FN1, ITGB1, GADPH, ENO1, HSPA4, HSPA8, HSP90AA1, HSP90AB1, ANXA2, A1BG, DNTM1, TSR1, RPS27, PPM1G, SMC2, LIG1, NCAPD2, POLD1, PRKDC, YBX1, ANK1, FTL, NCL, ITGB2, SERPINA7, HP, and A2M). The first 10 proteins (underlined) were shared also with non‐metastatic tumors. Label‐free quantitative proteomics identified 145 differentiated protein, 15 of which were enriched and 130 impoverished specifically in metastatic tumors. Evidence suggests that HSPA4, HSP90AB1, DNTM1, RPS27, FTL, NCL, A2M are implicated in the pathogenesis and progression of colorectal cancer, positioning them as potential prognostic biomarkers for the onset of metastasis.

**Conclusion:**

Combination of PIMS and NPOT coupled to label‐free quantitative proteomics point towards the distinct panitumumab mode of action in CRC patients and highlights specific proteins as prognostic biomarkers which need further validation in a bigger cohort and multicentric investigation, ideally involving patient registry follow up data.

**Novelty and Impact:**

This study presents an integrative molecular profiling strategy that combines PIMS, NPOT, and proteomics to uncover mechanistically relevant biomarkers of therapeutic response in colorectal cancer. By identifying an EGFR‐centered interactome and responder‐specific protein signatures, the research offers a novel approach to stratify panitumumab response and supports advancement of precision oncology in clinical settings.

## Graphical abstract

Integrated PIMS, NPOT, and proteomics analysis reveals an EGFR‐associated interactome and candidate biomarkers to stratify Panitumumab response in metastatic colorectal cancer.

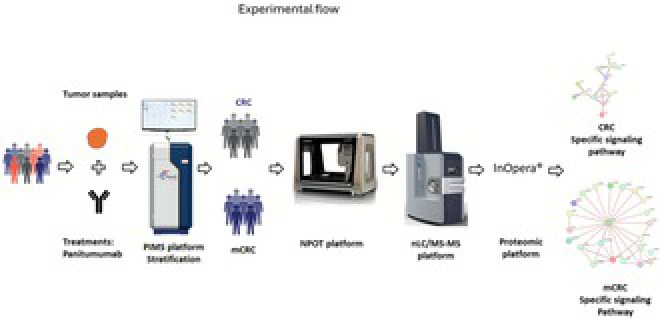

**Abbreviations**
A2M: alpha‐2‐macroglobulin
ACN: acetonitrile
BCA: bicinchoninic acid
CRC: colorectal cancer
DNTM1: DNA methyltransferase 1
EGFR: epidermal growth factor receptor
FA: formic acid
FTL: ferritin light chain
HBSS: Hanks' balanced salt solution
InOPERA: Inoviem Protein Ranking and Analysis
MMR: mismatch repair
MSI: microsatellite instability
NIR: near infra‐red
NPOT: Nematic Protein Organization Technic
NPV: negative predictive values
PASEF: parallel accumulation–serial fragmentation
PIMS: Physiological Intermolecular Modulation Spectroscopy
PPV: positive predictive values
RV: resonance volume
SDS‐PAGE: sodium dodecyl sulfate‐polyacrylamide gel electrophoresis
TMB: tumor mutational burden

## 1 Introduction

Colorectal cancer (CRC) remains a leading cause of cancer‐related morbidity and mortality worldwide, with metastatic disease presenting a major barrier to effective therapeutic intervention. While targeted therapies such as Panitumumab, a monoclonal antibody against the epidermal growth factor receptor (EGFR), have demonstrated clinical benefits in select patient populations, the lack of reliable predictive biomarkers continues to hinder precise treatment response monitoring [1, 2]. The inherent heterogeneity of CRC tumors and the emergence of resistance mechanisms further complicate treatment outcomes, underscoring the need for advanced molecular profiling approaches to optimize patient stratification and therapeutic decision‐making.

Recent advancements in high‐resolution proteomics and molecular interactome characterization have provided deeper insights into EGFR‐associated signaling pathways and resistance mechanisms [3, 4]. To leverage these advancements, this study utilized Physiological Intermolecular Modulation Spectroscopy (PIMS), a molecular resonance‐based approach that measures protein interaction dynamics and structural changes within tumors. PIMS enables label‐free, high‐sensitivity detection of tumor‐specific resonance patterns, making it a powerful tool for differentiating metastatic from non‐metastatic tumors and predicting therapeutic responses [5, 6]. In parallel, we applied Nematic Protein Organization Technic (NPOT), an unbiased label‐free interactome profiling method, which provides a global assessment of protein–protein interactions and signaling networks [7, 8], here comparing metastatic to non‐metastatic tumor's interactomes By integrating these cutting‐edge technologies, we aimed to overcome the limitations of conventional biomarker discovery approaches and identify distinct molecular signatures associated with Panitumumab responsiveness and CRC progression.

To achieve this, we systematically analyzed 21 CRC tumor resection samples from 20 patients, encompassing both primary tumors (*n* = 12) and intrahepatic metastases (*n* = 9), with a focus on the EGFR‐associated interactome and downstream signaling pathways. Our findings reveal a distinct proteomic landscape and molecular resonance profile distinguishing metastatic from non‐metastatic tumors, alongside putative prognostic biomarkers linked to CRC progression. This study provides new insights into Panitumumab's mechanism of action, identifies candidate biomarkers for therapy stratification, and underscores the potential of PIMS and NPOT as next‐generation tools in precision oncology. Future validation in larger, multi‐center cohorts will be essential to translating these findings into clinical applications, ultimately advancing personalized therapeutic strategies for CRC patients.

## 2 Materials and Method

Informed consent for the collection of surgically resected tissues was obtained from patients at least 7 days prior to scheduled elective procedures. Following resection, specimens were promptly harvested and transported on dry ice to the proteomics facility from the surgical site. Patient demographics, including age, sex, tissue origin and TNM classification are summarized in Table 1.

**TABLE 1:** Clinicopathological characteristics of colorectal cancer patients (*n* = 20), including both primary tumors and liver metastases. Listed variables include sex, age at surgery, analyzed tissue type, TNM classification, metastatic status and survival data used for correlation with molecular and proteomic analyses.

Sample number	Sex	Age at time of surgery	Analyzed tissue	Occurrence of liver metastasis	TNM classification/grading	Time until liver metastasis (months)	Death of patient	Time until death since diagnosis (months)	Time until death since primary resection (months)
1	Male	65	Liver metastasis	Yes	pT3 pN2b (2/18) M1a (Hep) R0 L1 G2	32	Yes	63	63
2	Male	60	Liver metastasis	Yes	pT4a pN2b (10/23) M1a (Hep) R0 L1 V1 Pn0	0	—	—	—
3	Male	61	Liver metastasis	Yes	pT3 pN2b (7/26) pM1 (Hep) L1 V1 Pn1 R0 G2	17	Yes	20	19
4	Female	70	Primary tumor	No	pT4 pN+	—	—	—	—
5	Female	70	Liver metastasis	Yes	pT3 pN1b (2+/25) L1 V0 Pn0 cM1 (Hep) G2	10	—	—	—
6	Male	65	Primary tumor	No	pT2bpN1 (1/21) G2 L1 V1 R0	—	—	—	—
7	Male	86	Primary tumor	No	pT2 pN0 (0/19) L0 V0 Pn0 R0 G2 MMRp	—	—	—	—
8	Male	58	Liver metastasis	Yes	pT4b pN2b (10/32), L1, V1	5	—	—	—
9	Male	73	Primary tumor	Yes	pT4a pN0 (0/63) pM1 (Hep, PER) L0 V1 Pn1 R0 G2	4	—	—	—
9	Male	73	Liver metastasis	Yes	—	4	—	—	—
10	Male	61	Primary tumor	Yes	pT4a pN2(12/18) pM1(HEP) L1 V1 Pn1 R2 G2	0	Yes	10	1
11	Female	75	Primary tumor	No	pT3 pN0 (0/32) L1 V0 Pn0 R0 G3	—	—	—	—
12	Female	72	Primary tumor	No	pT3 pN0 (0/23) L0 V0 Pn0 R0 G2	—	—	—	—
13	Male	58	Primary tumor	Yes	pT4a pN0 (0/21) L0 V0 Pn0 R1 G3	0	—	—	—
14	Female	52	Primary tumor	No	pT2 pN0(0/27) L0 V0 Pn0 R0 G1	—	—	—	—
15	Male	72	Primary tumor	No	pT3 pN0 (0/23) L0 V0 Pn0 R0 G2	—	—	—	—
16	Male	59	Liver metastasis	Yes	pT3a pN1 (1/15) L1 V0 Pn0 M0 R0	7	—	—	—
17	Male	81	Primary tumor	No	pT2 pN0 (0/31) L0 V0 Pn0 R0 G2	—	—	—	—
18	Male	61	Liver metastasis	Yes	cT3c N2 M0	20	—	—	—
19	Female	43	Liver metastasis	Yes	pT3 pN1a (1/39) L1 V1 pN0 pM1 R0 G2	9	—	—	—
20	Male	59	Primary tumor	No	pT4a pN2a (5/26) L1 V1 Pn1 R0 G2	—	—	—	—

### 2.1 Physiological Intermolecular Modification Spectroscopy

PIMS is a biophysical technology based on near infra‐red (NIR) spectroscopy and is able to measure changes in water molecule resonance within any biological sample and tissue [9]. Tissue water molecule resonance reflects the change in macromolecular conformation to their inherent resonance. The latter is amplified by modulating the temperature. These are suited for rapid non‐destructive water determination [10], all shifting a few nm to longer wavelengths (lower frequency) with strengthening of hydrogen bonding due to shifts from high‐density water (increasing collapsed structure) to low‐density water (increasing expanded structure) [5, 6, 11]. PIMS is therefore able to study protein–protein and protein–solvent interactions in multi‐component solutions. It provides individual real‐time dynamic fingerprints of total macromolecular assemblies in a tissue both at base line and in the presence and absence of exogenous molecules (drug or drug candidates, peptides, or proteins). It shows the patient's molecular capacity to respond to a drug substance and allows for discrimination between different subpopulations of patients regarding the capacity to respond to a specific therapy.

### 2.2 Base Line Analysis

Patient tumors biopsies (*n* = 20) (female *n* = 6, age 67 ± 12.97, male *n* = 14, age 64 ± 8.98) were broken by three cycles of fast freezing vapor of liquid nitrogen and slow thawing on ice before proceeding to mechanical homogenization using a glass potter. Protein concentration was determined using the Pierce BCA Protein Assay (ThermoFisher Scientific) according to the manufacturer's recommendation.

One microgram of total homogenate tumor protein was diluted in 10 μL of HBSS and placed in a PIMS well (volume = 10 μL/well). Then, the PIMS records the light absorption of the samples, as a function of wavelength and temperature: each tumor base line analysis consists of 700 steps from 450 to 1700 nm compared to HBSS alone (at room temperature), followed by a sample freezing (−20°C) and finally acontinuous sampling as the temperature rose from −20°C to 20°C. This was repeated four times independently. PIMS^Q8^ software was then used to integrate the spectra and calculate the resonance volume of each patient's tumor, allowing different tumors' molecular behavior to be distinguished.

### 2.3 Challenge With Panitumumab

One microgram of total homogenate tumor protein was diluted in 10 μL of HBSS and supplemented with 1 μg of panitumumab (volume = 11 μL/well). Then, the experiment was set up as for the base line analysis, with the exception that the sample was compared to panitumumab (1 μg)/HBSS (volume = 11 μL/well). PIMS^Q8^ software was then used to integrate the spectra and calculate the resonance volume of each patient's tumor in the presence of panitumumab, allowing different tumors' molecular behavior to be distinguished.

### 2.4 Nematic Protein Organization Technique

The NPOT is a label‐free method dedicated to the isolation and identification of specific macromolecular scaffolds directly from human tissues. The NPOT is based on the Kirkwood‐Buff molecular crowding and aggregation theory [12, 13]. It enables the formation and label‐free identification of macromolecular complexes (heteroassemblies) involved in physiological or pathological processes, including the analysis of protein–protein and protein‐ligand interactions in cells or human tissue, without disrupting the native molecular conformation.

Here, tumors from patients (*n* = 5) were prepared in the absence of any detergent by three cycles of fast freezing and slow thawing as described before. Tumors were thereafter mechanically broken using glass and Teflon potter subsequently. Protein concentration was determined using Pierce BCA Protein Assay (ThermoFisher Scientific) Then, one microgram of total protein extract from each tumor homogenate was put into the NPOT system with a large pH gradient of 5–9 prior to the addition of 1 μg of panitumumab, leading to heteroassemblies formation (examples in Figure 4). These heteroassemblies contain panitumumab primary and secondary targets, together with its native interactome, triggered by direct and indirect protein–protein interactions. The experiments have been performed in quadruplicate. Before LC–MS/MS analysis, heteroassemblies were solubilized directly in HBSS supplemented with 0.2% Triton X‐100.

### 2.5 Proteomic

After the addition of Laemmli buffer, stacking bands were run in sodium‐ dodecyl sulfate‐polyacrylamide gel electrophoresis (SDS‐PAGE) gels (BioRad). After gel band excisions, disulfide bonds were reduced for 1 h at 60°C by adding dithiothreitol to a final concentration of 10 mM and alkylated for 20 min in the dark by adding iodoacetamide to a final concentration of 30 mM. An overnight digestion at 37°C was performed by adding trypsin. Peptides were extracted (80% acetonitrile [ACN], 0.1% formic acid [FA]), dried using SpeedVac (ThermoSavant) coupled with the Refrigerated Vapor Trap (ThermoFisher Scientific), and diluted with 0.1% FA before mass spectrometry analysis. Each sample was then loaded onto an Acclaim PepMap 100 C18 HPLC column (20 × 0.1 mm, 5 μm particle size; ThermoFisher Scientific), one run per NPOT replicate. The peptides were separated on an Aurora UHPLC C18 Column with nanoZero fitting and CaptiveSpray Insert (250 mm × 75 μm, 1.6 μm particle size; IonOpticks). The solvent system consisted of 0.1% FA and 2% ACN in water (solvent A) and 0.1% FA in ACN (solvent B). The samples were loaded into the enrichment column at a constant pressure of 80 bars with solvent A. Elution of the peptides was performed at 50°C at a flow rate of 300 nL/min with a gradient of 2%–30% solvent B in 30 min.

The timsTOF Pro was operated in parallel accumulation–serial fragmentation (PASEF) mode. The CaptiveSpray was running at a spray voltage of 1.6 kV at 180°C. The trapped ion mobility spectrometry cycle was set to 1.88 s (ion mobility coefficient scan range: 0.7–1.25 V·s/cm^2^) and contained 1 MS and an average of 10 PASEF MS/MS scans. MS and MS/MS spectra were recorded from 100 to 1700 m/z. Exclusion time was set to 0.4 min.

The raw data obtained during nanoLC‐MS/MS analyses were converted into “.mgf” files using DataAnalysis and were interpreted using Mascot (Matrix Science). The databases were extracted from Swissprot (human taxon; ID 9606; 20,517 entries). All entries belonging to the taxonomy were extracted; common contaminants and reverse copies of all sequences were added thanks to the database toolbox of MSDA (https://msda.unistra.fr). Trypsin was selected as the cleavage enzyme and a maximum of one missed cleavage was allowed. Mascot results were loaded into the Proline software for validation (http://proline.profiproteomics.fr). For validating peptide spectrum matches, the following parameters were applied: a rank of 1, a minimum peptide length of 7 amino acids, and a minimal score of 25. In addition, a false discovery rate of 1% was applied to the protein set.

List of proteins was then analyzed using Inoviem Protein Ranking and Analysis (InOPERA) database which comprises the proteomics datasets of more than 250 NPOT projects and calculates the occurrence of given proteins in the entire database, or specific data sets matching defined criteria of species, organs, and so forth. Abundant protein hits that have been observed in the same tissue and/or same species are identified using this bioinformatic application. The global protein dataset was analyzed to highlight specific proteins for each pathology within a core network (using STRING network data resource).

### 2.6 Quantitative Proteomics

#### 2.6.1 Quantification

Raw data were processed using MaxQuant software (version 2.1.4). Peaks were assigned with the Andromeda search engine with trypsin/P specificity against an in‐house generated database containing all human entries extracted from UniProtKB‐Swissprot (6th of January 2023, 20,441 entries). The minimal peptide length required was seven amino acids and a maximum of one missed cleavage was allowed. One fixed modification was considered: carbamidomethyl (Cys) and several variable modifications: oxidation (Met) and acetylation (protein N‐term). The “match between runs” option was enabled. The maximum false discovery rate was set at 1% at peptide and protein levels with the use of a decoy strategy. Intensities were extracted from the ProteinGroup.txt file to perform further statistical analysis.

#### 2.6.2 Statistical and Differential Analysis

Protein intensities were loaded into Prostar software (v.1.34.4 and DAPAR v1.34.4) and data split into two conditions according to the clinical data (patient with metastasis or without metastasis). Contaminants, reverse proteins, and proteins only identified by site were removed. Proteins with at least 60% of non‐missing values, in at least one condition, were kept. Normalization of intensities was performed using quantile centering normalization with a 15% quantile. Missing values were imputed using the *det quantile* algorithm and a 2.5% quantile. Finally, a student *t*‐test was applied as well as Benjamini–Hochberg *p*‐value calibration. Differential analysis of the metastasis versus no metastasis conditions was performed, with a 1% *p*‐value filter.

### 2.7 Differential Expression Analysis of Panel Gene

RNA‐seq expression profiles and corresponding clinical data were obtained from the TCGA‐READ project (FPKM format, log_2_[FPKM + 1] transformation). The dataset included rectal adenocarcinoma samples classified as non‐metastatic (*n* = 130) and metastatic (*n* = 23) in the current download cohort. A targeted gene panel comprising ANK1, HSPA4, HSP90AB1, DNMT1, RPS27, FTL, NCL, and A2M was analyzed to compare expression patterns between the two groups. Group comparisons were performed using an independent‐samples *t*‐test when normality assumptions were met, or the Wilcoxon rank‐sum test otherwise, with statistical significance defined as *p* < 0.05.

## 3 Result

**FIGURE 1:**
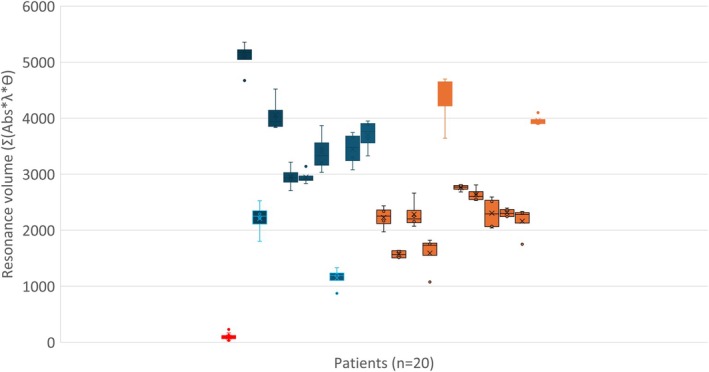
Showing CRC tumors resonance volume (RV) calculated from 4 independent PIMS experiments, for each patient (*n* = 20). Patients with metastasis (dark blue) have higher resonance volume than patients without (orange with dark blue lines). There are two outliers in patients with metastasis with low RV (dark blue with bleu lines) and two patients without metastasis with high RV (orange) PPV = 0.78 and NPV = 0.82.

Analysis of 20 CRC tumor samples, including both primary tumors and metastases, revealed distinct molecular signatures associated with metastatic progression and Panitumumab responsiveness. PIMS molecular resonance profiling achieved an 82% accuracy in differentiating metastatic (*n* = 9) from non‐metastatic (*n* = 7) tumors, with metastatic tumors exhibiting significantly higher resonance volumes (2948–5094) compared to non‐metastatic tumors (1076–2759) (Figure 1). This suggests that PIMS captures biomechanical or structural alterations associated with tumor progression, potentially reflecting differences in tumor rigidity, extracellular matrix composition, or receptor clustering dynamics, features known to correlate with cancer cell invasiveness and metastatic potential [14].

**FIGURE 2:**
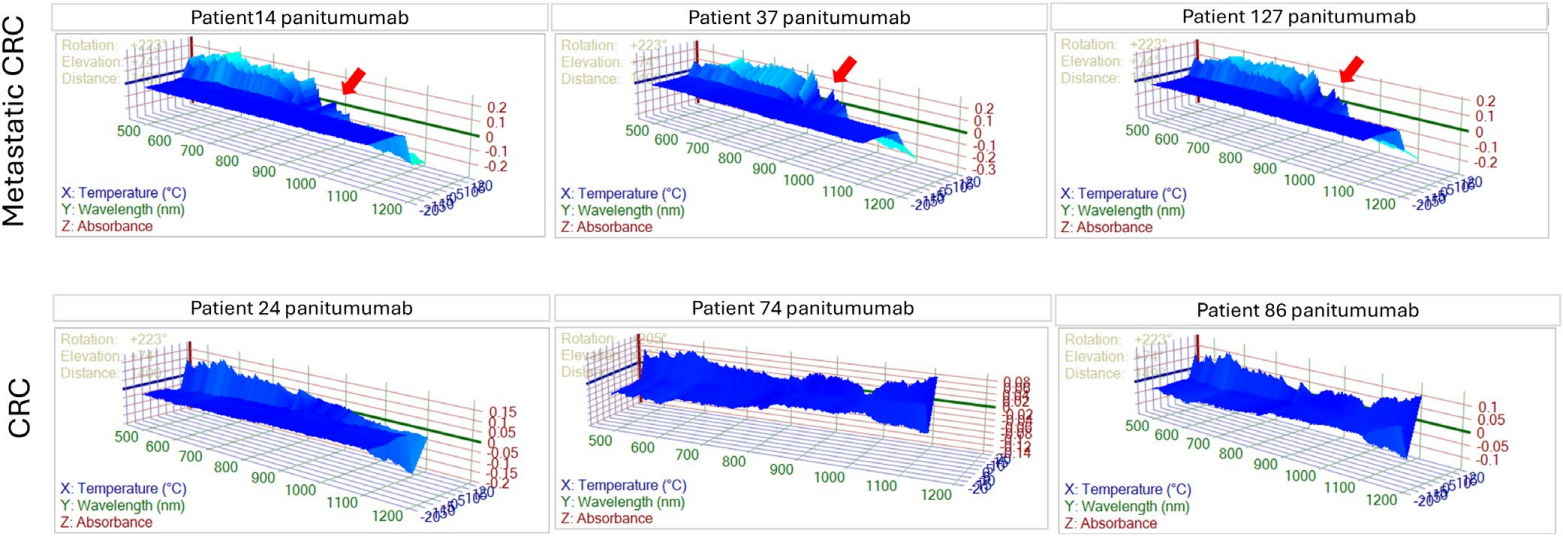
Showing PIMS results on CRC tumor resonance spectra where wavelength (nm), temperature (°C), and absorbance are plotted in *X*, *Y*, and *Z* respectively. The upper panel shows 3 patients with metastatic colorectal cancer (mCRC), and the lower panel shows 3 patients with non‐metastatic colorectal cancer (CRC) challenged with panitumumab.

**FIGURE 3:**
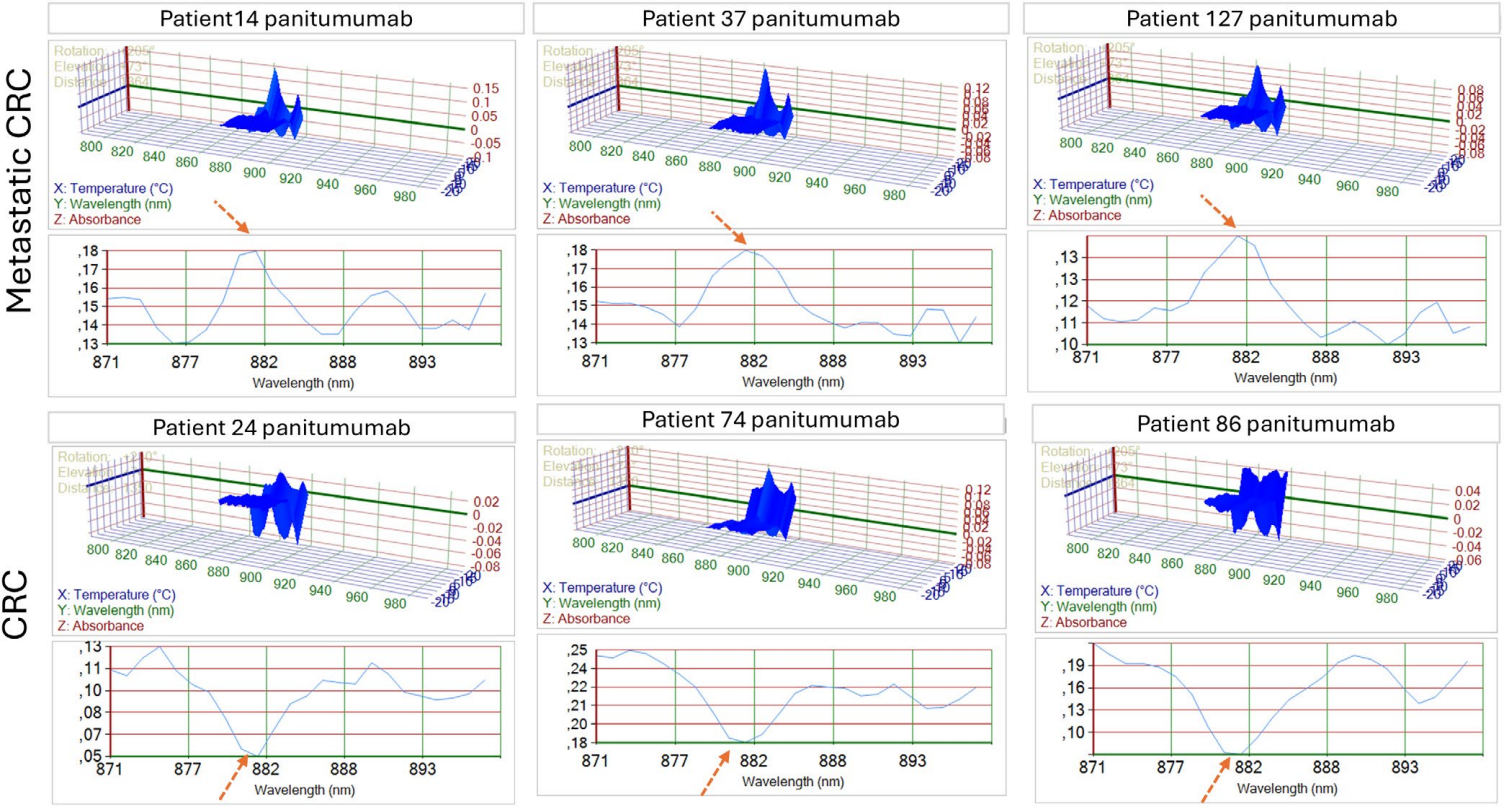
Showing zoom (875–900 nm) PIMS results on tumor resonance volume (RV) where wavelength (nm) and the sum of absorbances for all temperature intervals are plotted in *X*, and *Y*, respectively. CRC tumor resonance spectrum is inversed at 880 nm between mCRC and CRC challenged with panitumumab (red dotted arrow).

Further EGFR‐targeted PIMS screening, post‐blinding, identified six Panitumumab responders and six non‐responders, providing a refined molecular stratification (Figures 2 and 3). There is a higher molecular resonance 870–900 nm in mCRC challenged with panitumumab compared to CRC patients (red arrow in Figure 3).

**FIGURE 4:**
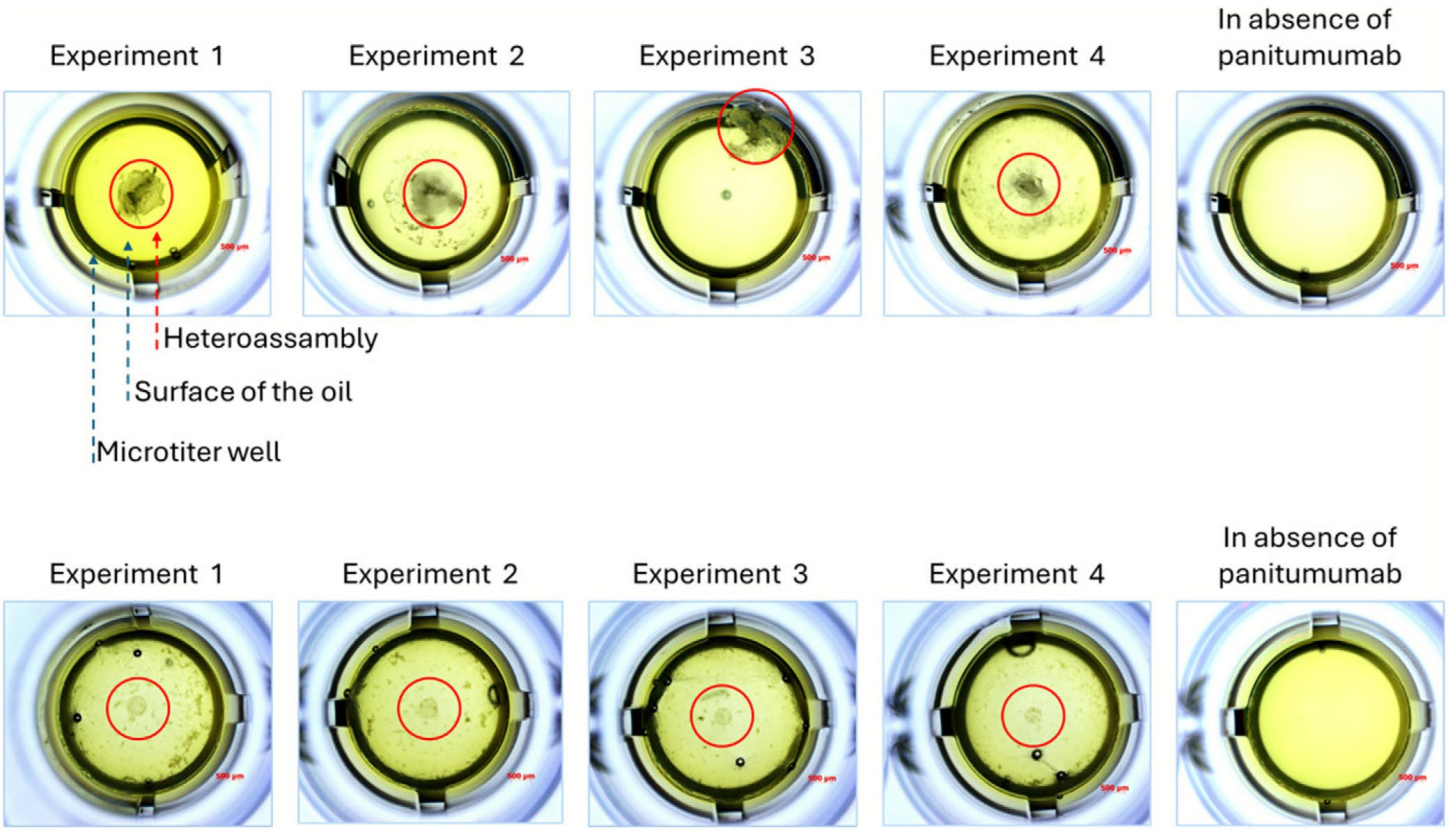
Representative hereoassemblies from NPOT experiments with a metastatic (upper panel) or non‐metastatic CRC tumor (lower panel) in presence of panitumumab. The formed heteroassemblies of tumor proteins specifically in presence of panitumumab are circled in red. In absence of panitumumab no heteroassembly is formed. The experiments have been performed in quadruplicate for each tumor.

**FIGURE 5:**
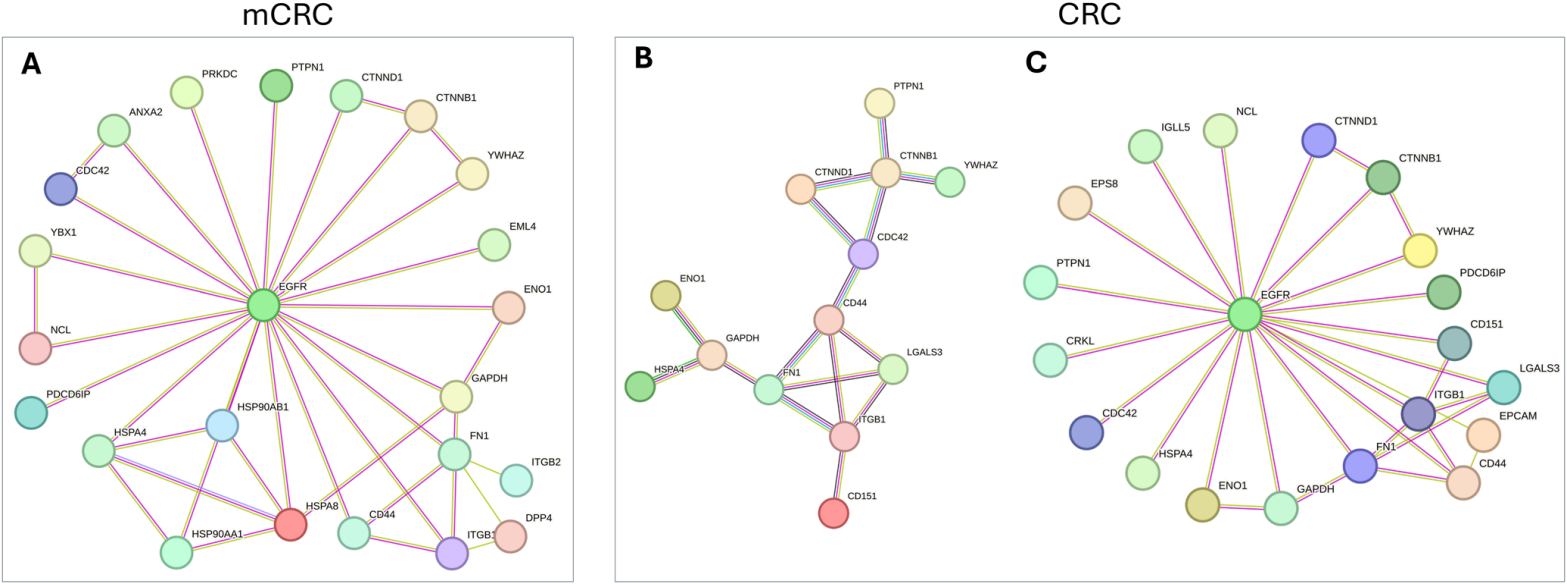
String network post InOPERA analysis with specific and overlapping proteins for mCRC and CRC groups. EGFR was only identified in mCRC patients. The EGFR and its related protein interactome are shown (A). The isolated interactome for CRC patients is shown (B). Note the change in their interaction when EGFR is added manually to the STRING network (C).

Then, NPOT analysis of panitumumab interactome in metastatic versus non‐metastatic tumors demonstrated that EGFR was exclusively present in metastatic tumors, suggesting a distinct EGFR‐driven signaling landscape in advanced CRC (Figures 4 and 5). More precisely, the metastatic EGFR interactome consisted of 34 proteins (Figure 5A), with 10 proteins (PTPN1, CTNNB1, CTNND1, YWHAZ, CD44, FN1, ITGB1, GADPH, ENO1, HSPA4) shared with non‐metastatic tumors (Figure 5B), while the remaining 24 proteins were uniquely present in metastatic tumors. The differential interactome composition indicates a functional shift in EGFR signaling that may drive increased tumor cell adhesion (CD44, ITGB1, FN1), metabolic adaptation (GAPDH, ENO1) and stress response (HSPA4, HSP90AB1), consistent with mechanisms observed in therapy‐resistant CRC phenotypes [15].

**FIGURE 6:**
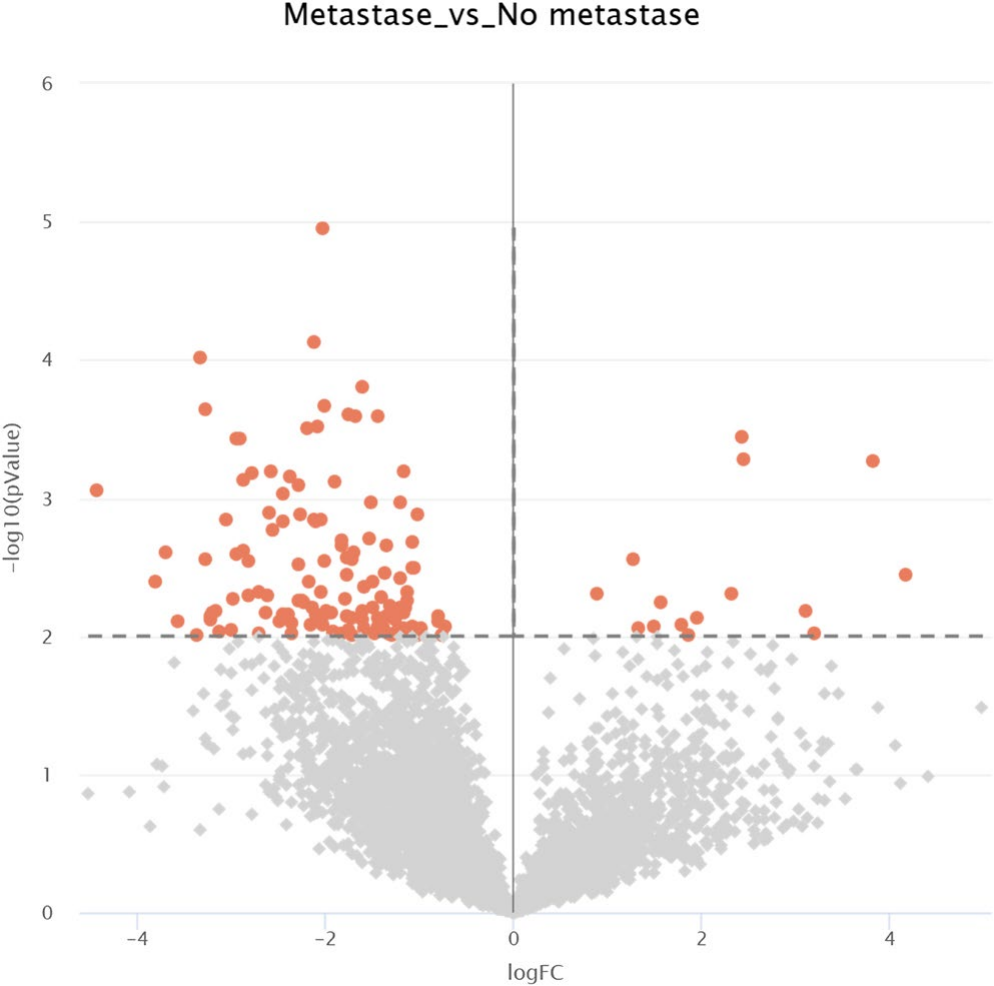
Volcano plot after quantitative analysis of proteins isolated by NPOT. All proteins above the threshold (dotted line) are significantly impoverished (left) or enriched (right) in mCRC.

**FIGURE 7:**
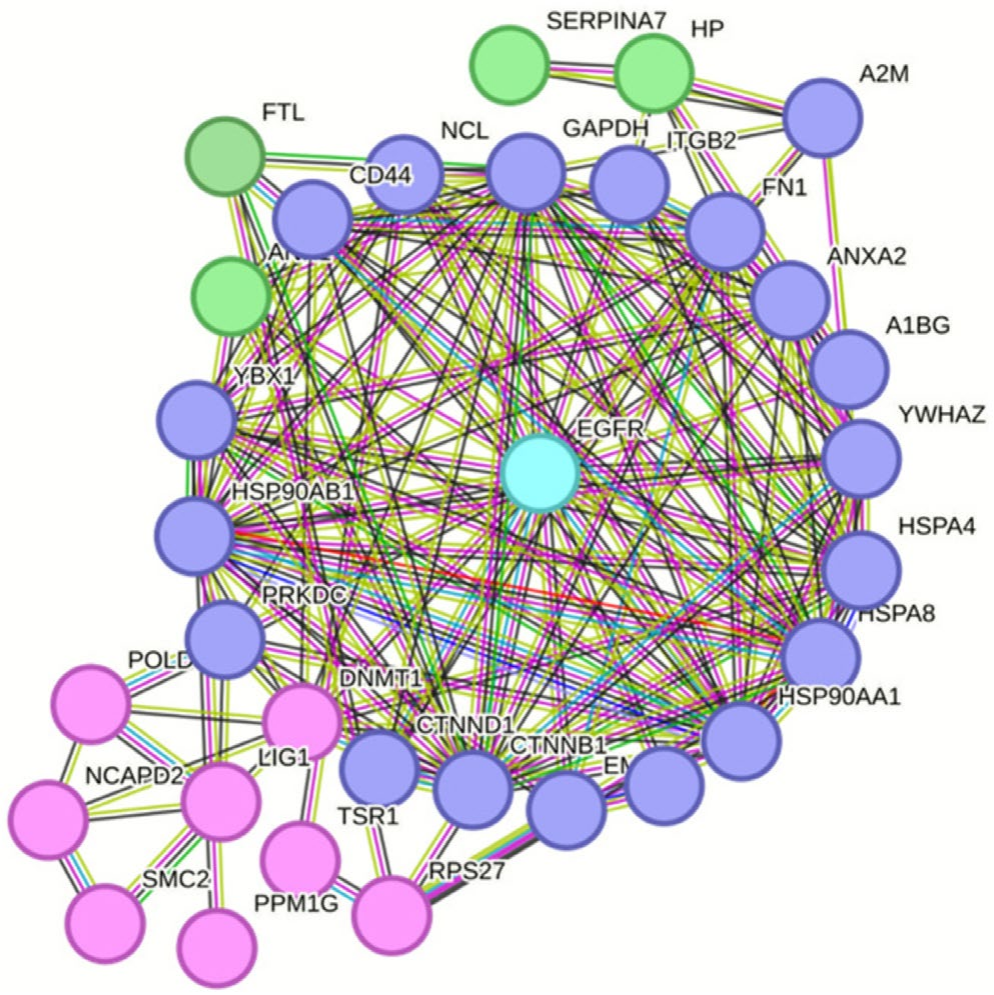
Merge of STRING networks post InOPERA analysis regrouping proteins specific for mCRC and label free quantitative analysis: Proteins in green are enriched and in pink are impoverished in mCRC tumors.

**FIGURE 8:**
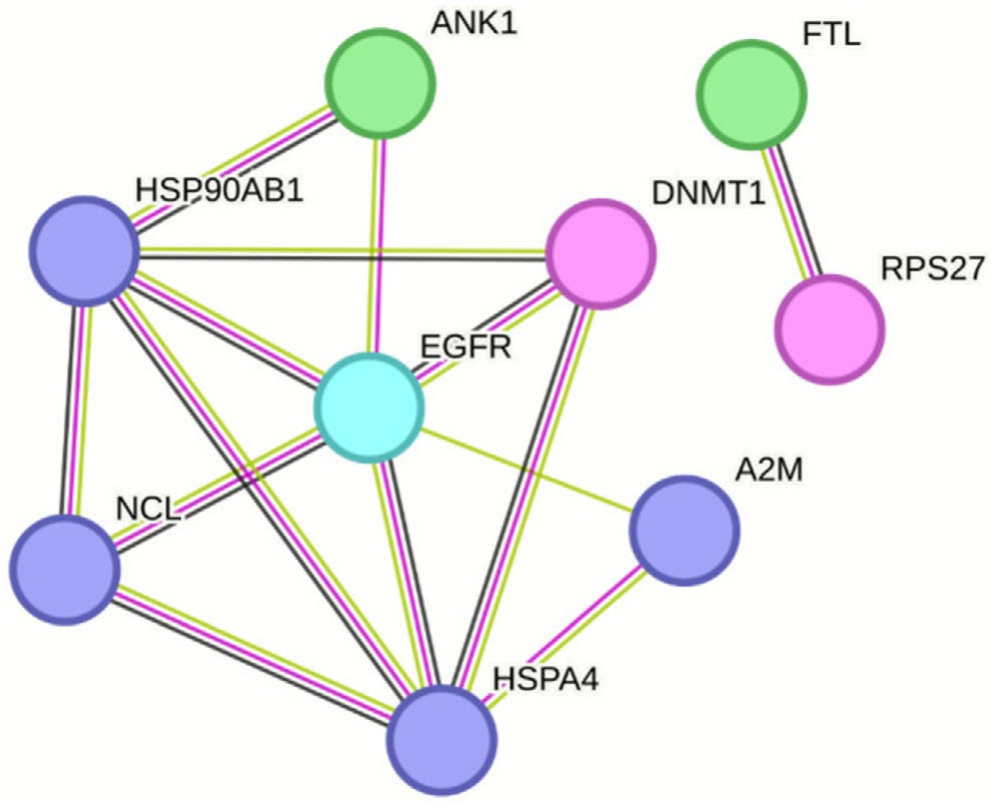
The near interactome of EFGR with the 8 identified proteins specifically with panitumumab in mCRC patients. These proteins are known to be involved in the maintenance of the disease. Proteins in green are enriched, and proteins in pink are impoverished in mCRC tumors.

Label‐free quantitative proteomics further identified 145 differentially expressed proteins, with 15 enriched and 130 impoverished in metastatic tumors (Figure 6). Integration of these results to the previous STRING network highlight a near interactome of EGFR (Figures 7 and 8). Notably, ANK1, HSPA4, HSP90AB1, DNMT1, RPS27, FTL, NCL and A2M emerged as key molecular players, each previously implicated in CRC pathogenesis and progression. HSPA4 and HSP90AB1, members of the heat shock protein family, are known to promote protein folding, cellular stress responses, and resistance to apoptosis, potentially enhancing CRC cell survival in the metastatic niche [16]. DNTM1 (DNA methyltransferase 1) is a critical epigenetic regulator, frequently upregulated in CRC, where it contributes to tumor suppressor gene silencing and chemotherapy resistance [17]. RPS27, a ribosomal protein, has been linked to tumor growth and increased metastatic potential via its role in mRNA translation and p53 modulation [18]. FTL (ferritin light chain) and A2M (alpha‐2‐macroglobulin) are metabolic and immune‐modulating proteins, respectively, which have been correlated with CRC progression, inflammation‐driven metastasis, and resistance to targeted therapies [19, 20].

**FIGURE 9:**
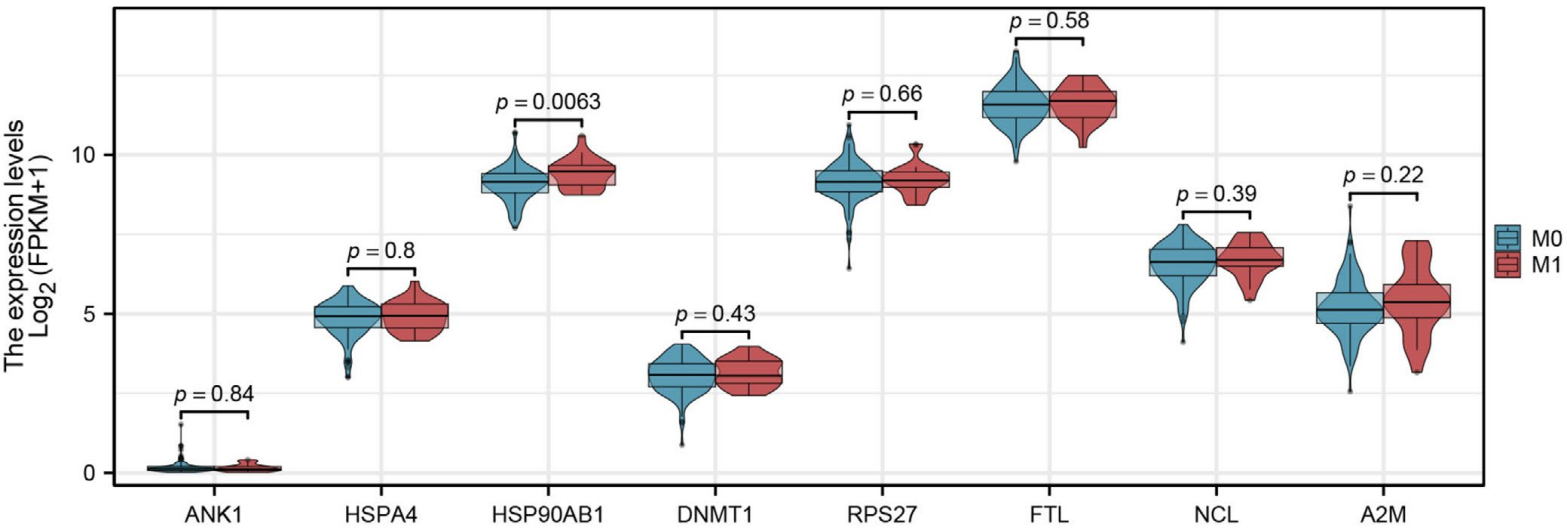
Violin plots showing transcript levels of eight candidate genes in primary (M0) and metastatic (M1) rectal cancer. HSP90AB1 was significantly upregulated in metastatic tumors (*p* = 0.0063*), supporting its proteomic identification as a key marker of metastatic progression (* indicates *p* < 0.05 as statistical significance).

Integration of the proteomic data with public transcriptomic resources further supported the biological relevance of the identified proteins (Figure 9). Among the eight key candidates linked to the EGFR‐centered interactome, HSP90AB1 showed a clear and statistically significant elevation in metastatic tumors, while the others displayed similar but non‐significant upward trends. This expression pattern reinforces the proteomic observation that HSP90AB1 is preferentially enriched in the metastatic setting. Its overexpression in independent transcriptomic datasets highlights a consistent molecular signature of metastatic adaptation. Functionally, HSP90 acts as a central molecular chaperone that stabilizes multiple oncogenic proteins, including EGFR, thereby promoting sustained signaling, stress tolerance, and therapeutic resistance in colorectal cancer [21, 22].

## 4 Discussion

The oncology in general is facing difficulties both in diagnostics and in treatment. The diagnostics are limited to the phenotype and if not all, but majority of cases fail to describe the endotype. Good knowledge on endotype increases efficacy of therapy and it opens for precision treatment that is based on right patient, right target, and right therapeutic window. The latter tends to be more efficient than the current try, and error based therapeutic approach.

Comparisons of patients using omics have brought at best the descriptive differences and by far no mechanistic explanation. Better to say, we have been making therapeutic decisions based on correlation rather than causalities. One powerful attempt to reach causality and overcome the difficulties is to understand the mode of action of the treatment in responder cohort and identify the underling molecular mechanism of the response. This would on the one hand help to identify success predictors and avoid missing the therapeutic window. Through this study, we have tried to explore such an approach by study the molecular mechanisms of response to panitumumab in CRC patients.

We know that the tumor microenvironment is not limited to the proteins identified in this study. The tumor structure beside its complexity is subjected to continuous remodeling. One of the major causes of the latter is iatrogenic as the tumor adapts itself to the treatment. Most of the omics explorations are based on comparison between two groups with two different phenotypes, whether they are healthy versus CRC or metastatic versus non‐metastatic. In fact, a simple comparison between the two groups would be informative in patients with the same endotype yet not same phenotype. Contrary to classical approach, in this study, instead of comparing the two different CRC groups with and without metastasis we sought to identify the molecular differences between the two groups regarding panitumumab. In our study, patient endotypes remain also unknown. To overcome the latter, we explored molecular mechanism in response to EGFR inhibitor in patients treated with panitumumab. This way the obtained information would be in relation to the clinical efficacy of the antibody that is directly related to the responder patient group. This would provide valuable information to decipher the protein interactomes between panitumumab sensitive and non‐sensitive.

We were able to register EGFR and panitumumab target engagement in PIMS and isolated the related signaling pathway using NPOT in metastatic CRC patients. We identified several proteins known to be involved in the maintenance and progression of the disease. Although the study cohort is limited, the identified proteins are in line with published literature. The presence of ANK1, HSPA4, HSP90AB1, DNMT1, RPS27, FTL, NCL and A2M in the metastatic CRC patients is highly evocative yet we do not know if they are acting in synergy with each other and if so which one of them would be the key protein beside the EGFR in panitumumab efficacy. Two proteins FTL and PRS27 are not known to interact directly or be a part of the EGFR interactome although they play a role in mCRC. Their presence in mCRC is differentiated by enriched FTL and impoverished PRS27. Their role in mCRC and panitumumab efficacy remains elusive at this point.

Our approach utilizing PIMS and NPOT represents a significant advancement in overcoming the limitations of current predictive models for immune therapy resistance in mismatch repair (MMR) intact CRC. Conventional predictive strategies, which primarily rely on microsatellite instability (MSI) status, PD‐L1 expression and tumor mutational burden (TMB), often fail to capture the dynamic nature of protein interactions and adaptive signaling networks that drive immune evasion MMR‐proficient tumors [23, 24]. By integrating high‐resolution proteomics with molecular resonance profiling, our method enables a more comprehensive characterization of the tumor microenvironment, uncovering metabolic reprogramming, stress response pathways and post‐translational modifications that play pivotal roles in tumor immune escape and therapy resistance. These findings further reveal a distinct molecular architecture in metastatic CRC, characterized by enhanced EGFR signaling, metabolic reprogramming and stress adaptation mechanisms, potentially contributing to Panitumumab resistance. The identification of specific proteomic signatures distinguishing responders from non‐responders highlights novel prognostic and predictive biomarkers that warrant further validation. Future large‐cohort and longitudinal studies will be essential to refine these molecular insights and establish their clinical utility in guiding personalized treatment strategies for CRC patients.

## Author Contributions

**Angelique Quartier:** investigation, data curation, writing – original draft, writing – review and editing, methodology, software, visualization, conceptualization. **Ahmed Y. Sanin:** investigation, data curation, writing – original draft, writing – review and editing, methodology, visualization, conceptualization. **Julia Nagelschmitz:** investigation, data curation, writing – review and editing. **Justine Schneider:** investigation, data curation, writing – original draft, writing – review and editing, methodology. **Wenjie Shi:** writing – review and editing, investigation, data curation, visualization, validation. **Thomas Wartmann:** methodology, writing – review and editing, resources. **Maximilian Dölling:** writing – review and editing, methodology, resources. **Frederike Stelter:** methodology, resources, writing – review and editing. **Mihailo Andric:** methodology, writing – review and editing, resources. **Roland S. Croner:** writing – review and editing, project administration, supervision, methodology, resources. **Pierre Eftekhari:** writing – original draft, writing – review and editing, methodology, resources, investigation, supervision, project administration, conceptualization. **Ulf D. Kahlert:** writing – review and editing, project administration, supervision, methodology, resources, investigation, funding acquisition, conceptualization.

## Ethics Statement

The study was approved by ethical commission of medical faculty of the Otto von Guericke University Magdeburg, Germany under registry 330/01 and 46/22.

## Consent

Written informed consent was obtained for anonymized patient information to be published in this article.

## Conflicts of Interest

P.E. is CSO, A.Q. and J.S. are employees of Inoviem Scientific, Illkirch Graffenstaden, France. Other authors declare no conflicts of interest.

## Data Availability

All data can be obtained from author A.Q. and A.Y.S. upon reasonable request.
